# Disruption of the with no lysine kinase–STE20-proline alanine-rich kinase pathway reduces the hypertension induced by angiotensin II

**DOI:** 10.1097/HJH.0000000000001554

**Published:** 2017-09-14

**Authors:** Luz G. Cervantes-Perez, Maria Castaneda-Bueno, Jose V. Jimenez, Norma Vazquez, Lorena Rojas-Vega, Dario R. Alessi, Norma A. Bobadilla, Gerardo Gamba

**Affiliations:** a Department of Pharmacology, Instituto Nacional de Cardiología Ignacio Chávez; b Department of Nephrology and Mineral Metabolism, Instituto Nacional de Ciencias Médicas y Nutrición Salvador Zubirán, Mexico City, Mexico; c MRC Phosphorylation and Ubiquytilation Unit, Dundee University, Dundee, United Kingdom; d Molecular Physiology Unit, Instituto de Investigaciones Biomédicas, Universidad Nacional Autónoma de México, Mexico City, Mexico

**Keywords:** aldosterone, distal convoluted tubule, salt transport, thiazide, with no lysine kinase 4

## Abstract

**Objective:**

The hypertensive effect of angiotensin II (AngII), a peptide hormone, is dependent on its intrarenal actions and the activation of the renal Na–Cl cotransporter (NCC), by AngII requires integrity of the with no lysine kinase/STE20-proline alanine-rich kinase (WNK/SPAK) signaling pathway. Here, we analyzed if the integrity of the WNK/SPAK pathway is required for AngII infusion to induce arterial hypertension.

**Methods:**

We tested the effect of AngII or aldosterone administration on the blood pressure and on pNCC/NCC ratio in SPAK^T243A/243A^ knock-in mice in which the kinase and thus NCC cannot be activated by WNK kinases. AngII or aldosterone was infused at 1440 or 700 μg/kg per day, respectively, for 14 days using osmotic minipumps. The aldosterone-treated mice were exposed to NaCl drinking water (1%) during the hormone administration. The arterial blood pressure was assessed using radiotelemetry.

**Results:**

We observed that in the SPAK knock-in mice, the AngII-induced hypertensive effect was significantly reduced and associated with an absence of AngII-induced NCC phosphorylation. In contrast, the hypertensive effect of aldosterone was enhanced and was related with an increased response to amiloride, but not to thiazide-type diuretics, without a significant increase in NCC phosphorylation.

**Conclusion:**

Our data suggest that AngII-induced hypertension requires, at least partly, NCC activation via the WNK/SPAK signaling pathway, whereas aldosterone-induced hypertension depends on epithelial sodium channel activation in a WNK/SPAK-independent manner. SPAK knock-in mice emerge as a useful model to distinguish between the effects of AngII and aldosterone on distal nephrons.

## Abstract 2

Supplemental Digital Content is available in the text

## INTRODUCTION

The arterial hypertension (AH) induced by angiotensin II (AngII) infusion depends on the intrarenal action of this hormone. The absence of AT1 exclusively in the kidney precludes the increase in blood pressure (BP) induced by AngII infusions using osmotic minipumps [1]. In the wild-type mice, this increase is associated with increased salt retention [2]. The absence of the intrarenal expression of angiotensin-converting enzyme also precludes the AngII-induced hypertension, suggesting that infused AngII induces the activation of the intrarenal renin angiotensin system; this activation then promotes salt retention and hypertension [3]. In addition, it has been shown that the salt retention and hypertension associated with AngII infusion is caused by increased salt reabsorption in the distal convoluted tubule (DCT) and the collecting duct [4,5].

The major salt transport pathway in the DCT is the thiazide-sensitive Na–Cl cotransporter (NCC). The NCC activation is associated with the phosphorylation of key threonine residues located in its amino terminus [6] by the Ste20-related proline–alanine-rich kinase (SPAK) [7]. In turn, this kinase is activated by the with no lysine kinases (WNKs) through WNK-induced phosphorylation of the threonine 243 and serine 383 of SPAK [8]. Mutation of threonine 243 in the SPAK^T243A/T243A^ knock-in mice (SPAK-KI) precludes the activation of SPAK by WNKs and results in a decreased effect of SPAK on the NCC; these mice display Gitelman-like phenotype, which is associated with a basal reduction of NCC expression and phosphorylation levels [9]. In addition, the Mendelian disease known as familial hyperkalemic hypertension (FHH) or pseudohypoaldosteronism type II is caused by mutations in two different serine/threonine kinases, WNK1 and WNK4, and in two genes encoding the proteins KLHL3 and CUL3 that form a ring-type E3-ubiquitin ligase that targets WNK kinases for ubiquitylation and degradation [10–12]. Mutations in any of these genes result in increased expression of WNKs, which causes increased activity of the NCC via activation of the SPAK-induced phosphorylation of this cotransporter [13,14]. We have previously shown *in vitro* and *in vivo* that the presence of WNK4 is required to achieve the NCC activation by AngII [15,16], which modulates WNK4 activity and phosphorylation via a protein kinase C (PKC)-related mechanism [17]. These observations strongly suggest that the AngII effect on the NCC requires the integrity of the WNK4–SPAK–NCC pathway. Here, we present evidence that AngII-induced AH is, at least partly, dependent on the activation of the NCC by the WNK–SPAK pathway.

## METHODS

### Experimental protocol

SPAK-KI mice were previously generated and characterized [9]. The experimental protocols were conducted in 12–16-week old (approximately 25 g) male SPAK-KI mice and their wild-type littermates, which were bred and maintained in the animal facilities of our institution. All experiments were conducted according to the Guide for the Care and Use of Laboratory Animals and were approved by the Animal Care Use Committee at our Institutions. Four separate experimental designs were followed: first, for telemetry studies SPAK-wild-type (SPAK-WT) and SPAK-KI mice were implanted and used as their own controls before and after AngII or Aldosterone infusion (*n* = 5). Second, for western blot analysis, SPAK-WT and SPAK-KI mice were infused with vehicle and compared with SPAK-WT and SPAK-KI mice infused with AngII or aldosterone (*n* = 5). BP was monitored by radiotelemetry to confirm the hypertensive effects of drugs. Third, for determining diuretic response, a single injection with hydrochlorothiazide or amiloride on SPAK-WT and SPAK-KI mice infused with AngII or aldosterone was administrated at 13th day of infusion (*n* = 5). Fourth, for studying diuretic effect on BP by radiotelemetry, a single doses of amiloride on SPAK-WT and SPAK-KI mice infused with AngII or aldosterone and with sensor implanted was injected at the 13th day of infusion (*n* = 5).

### Radiotelemetry

The mice were anesthetized using LEI Medical Table Top Anesthesia Machine for isofluorane (4% for induction and 2.5% for maintenance) (LEI Medical, Portland, Oregon, USA). An incision was made in the front of the neck, and the carotid artery was separated from the jugular vein and the vagus nerve. After the artery was ligated near the head using 3–0 silk sutures, a small incision was made with a needle, and the tip of the catheter of the transmitter (model PA-C10; Data Science International, St. Paul, Minnesota, USA) was introduced into the vessel and pushed until it was close to the aortic arch. The catheter was fastened using the distal silk. To place the transmitter, a subcutaneous pocket was made. The neck incision was sutured, and the mice were allowed to recover completely.

### Aldosterone and angiotensin II infusion

One week after the implantation of the radiotelemetry catheter and after 3-day 1-h of basal BP measurements, subcutaneous osmotic minipumps (model 1002; Alzet, Cupertino, California, USA) were implanted for the infusion of AngII (Sigma-Aldrich, St Louis, Missouri, USA) at 1440 μg/kg per day or aldosterone (Sigma-Aldrich) at 700 μg/kg per day for 14 days. The aldosterone-infused animals were provided with 1% saline solution as drinking water from days 0 to 14. The BP was measured using radiotelemetry (Dataquest A.R.T. system and PhysiolTel Receivers; Data Science International) every day at 1000 h for at least 1 h, with intervals of 5 s for each determination, giving 550 measures approximately for each mouse. During the 48-h period of continuous basal BP measurements, we determined the optimum time frame for reliable measurements, which was established for 1 h at 1000 h.

### Na–Cl cotransporter expression and phosphorylation

At the end of the infusion period, the mice were sacrificed, and kidneys from each group were homogenized in lysis buffer containing the following: 50 mmol/l Tris–HCl (pH 7.5), 1 mmol/l ethylene-bis(oxyethylenenitrilo)tetraacetic acid, 1 mmol/l ethylenediaminetetraacetic acid, 50 mmol/l sodium fluoride, 5 mmol/l sodium pyrophosphate, 1 mmol/l sodium orthovanadate, 1% (wt/vol) Nonidet P-40 (Sigma-Aldrich), 0.27 mol/l sucrose, 0.1% (vol/vol) 2-β**-**mercaptoethanol and protease inhibitors (Complete tablets; Co-Ro Roche, Sigma-Aldrich). Sixty micrograms from each homogenate were resolved into 10% SDS–PAGE and transferred for 1 h to polyvinylidene difluoride membranes. Membranes were blocked in 10% skim milk and incubated overnight with sheep antipNCC (T60) antibody, and after stripping, they were incubated with anti-NCC antibody, both produced by Dario Alessi from Phosphorylation Research Unit (Dundee, Scotland, United Kingdom) that were previously used and characterized by our group [7,18] and anti β-actin (Santa Cruz Biotechnology Inc, Dallas, Texas, USA) antibodies. For densitometric analysis purpose, total NCC and pNCC were normalized with β-actin, then, total pNCC/NCC ratio was calculated.

### Diuretic challenge

Another group of five SPAK-WT and five SPAK-KI mice were infused with AngII or aldosterone/NaCl 1% for 14 days. On ninth day, the mice were placed in metabolic cages for 3 days for acclimation. On 12th day, urine samples were collected for measuring basal sodium excretion. The next day (13th day), a single intraperitoneal (i.p.) injection of amiloride (Sigma-Aldrich) (5 mg/kg) or hydrochlorothiazide (Sigma-Aldrich) (50 mg/kg) was applied, and urine was collected 4 or 6 h, respectively, after diuretic injection and until 24 h for urinary sodium determination. Both collections were independent. Urine volume at 4 and 6 h was enough to measure electrolytes because we administrate a diuretic.

### Diuretic effect on blood pressure in angiotensin II and aldosterone-infused-mice

Different groups of five SPAK-WT and five SPAK-KI mice were implanted with radiotelemetry sensors and infused with AngII or aldosterone and 1% NaCl in drinking water. After 13 days of infusion, the mice were challenged with a single injection of amiloride (5 mg/kg). BP was measured continuously for 2 h before and 12 h after amiloride injection.

### Plasma and urinary determinations

Plasma and urinary samples were diluted 1 : 1 with distilled water and placed into an autoanalyzer (Technicon RA-1000; Bayer, Tarrytown, New York, USA) for sodium, potassium and creatinine determinations.

### Statistics

All values represent the mean and the standard error for each experimental value. One-way or repeated measurements analysis of variance were performed to analyze the difference between the groups. The data were significant for *P* less than 0.05.

## RESULTS

### The hypertensive effect of angiotensin II is reduced in Ste20-related proline–alanine-rich kinase-knock-in mice

**FIGURE 1:**
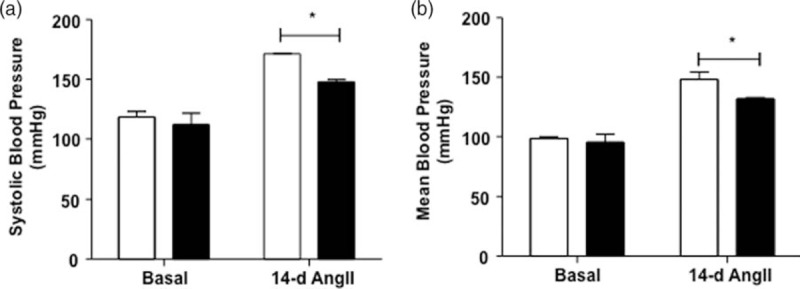
The arterial hypertension induced by angiotensin II infusion is blunted in Ste20-related proline–alanine-rich kinase-knock-in mice. (a) SBP and (b) mean blood pressure. Graphs represent SBP and mean blood pressure average of 3-day 1-h measures before angiotensin II infusion (basal) and the average of 12th, 13th and 14th-day 1-h after angiotensin II infusion (14-day) of wild-type (white bars) and Ste20-related proline–alanine-rich kinase-knock-in (black bars) mice. Blood pressure was assessed using radiotelemetry every day from 100 to 1100 h, during 17 days (before and after angiotensin II infusion). Each bar represents the mean ± standard error of approximately 550 measures of each mouse. *n* = 5 mice for each group. ^∗^*P* less than 0.05 wild-type vs. Ste20-related proline–alanine-rich kinase-knock-in.

Figure 1 shows the average of SBP (Fig. 1a) and mean BP (Fig. 1b) values observed during the 3-day 1-h previous to AngII infusion at baseline and in the last 3 days of AngII administration by the osmotic minipump (days 12, 13 and 14). In the basal period, the SBP was 118 ± 4.6 and 112 ± 9.5 mmHg and the mean BP was 98 ± 1.6 and 95 ± 7.2 mmHg in the wild-type and SPAK-KI mice, respectively. The differences were NS. Thus, we did not observe the difference in BP in the SPAK-KI mice as reported previously by Rafiqi *et al.*[9]. In that work, however, the mean BP observed in the SPAK-KI mice was similar to our data, around 96 mmHg, while the mean BP of control mice was higher, around 105 mmHg.

As depicted in Fig. 1, at the end of the AngII infusion period, the SBP and mean BP were significantly higher in the wild type than in SPAK-KI animals. SBP were 171 ± 0.3 and 147 ± 2.0 mmHg and mean BP were 148 ± 6.1 and 131 ± 1.0 mmHg for wild-type and SPAK-KI mice, respectively. The increment of BP in wild-type animals was about 50 mmHg, whereas in the SPAK-KI mice was about 35 mmHg. Thus, BP increase in SPAK-KI mice was about 35% lower than in wild-type mice.

### The Na–Cl cotransporter response to angiotensin II is abrogated in Ste20-related proline–alanine-rich kinase-knock-in mice

**FIGURE 2:**
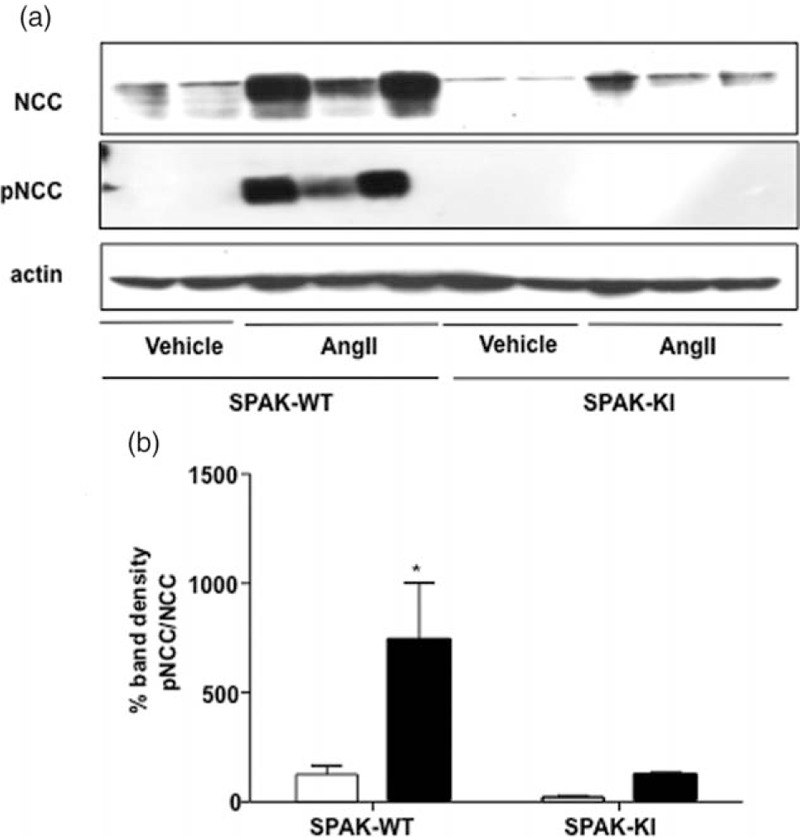
Angiotensin II infusion induced an increase in Na–Cl cotransporter expression and phosphorylation in wild-type, but not in Ste20-related proline–alanine-rich kinase-knock-in mice. (a) Representative western blot for total Na–Cl cotransporter, phosphor-Na–Cl cotransporter and β-Actin from proteins extracted from the kidney of wild-type or Ste20-related proline–alanine-rich kinase-knock-in mice infused with vehicle or angiotensin II, as stated. (b) Densitometric analysis of two independent western blot for total Na–Cl cotransporter and phosphor-Na–Cl cotransporter expressed as the pNa–Cl cotransporter/total Na–Cl cotransporter ratio after β-actin normalization. Open bars indicate vehicle infusion and closed bars indicate angiotensin II infusion. ^∗^*P* less than 0.0001 vs. all other groups.

As previously shown [9], the NCC basal expression and phosphorylation levels are lower in SPAK-KI mice than in their corresponding littermates. We analyzed the effect of vehicle or AngII infusion on NCC expression and phosphorylation levels (Fig. 2). AngII induced a significant increase in the pNCC/NCC ratio in the wild-type mice. In contrast, no effect was observed in the SPAK-KI mice. These observations suggest that NCC phosphorylation and the hypertensive effect of AngII are blunted in the kidneys of the SPAK-KI mice.

### The hypertensive effect of aldosterone is increased in Ste20-related proline–alanine-rich kinase-knock-in mice

**FIGURE 3:**
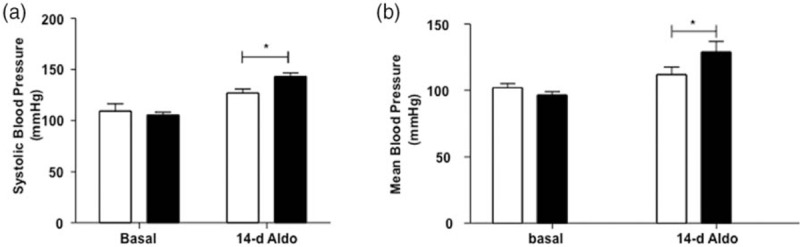
The effect in blood pressure induced by aldosterone infusion was higher in Ste20-related proline–alanine-rich kinase-knock-in mice. (a) SBP and (b) mean blood pressure. Graphs represent SBP and mean blood pressure average of 3-day 1-h measures before aldosterone infusion (basal) and the average of 12th, 13th and 14th-day 1-h after aldosterone infusion (14-day) of wild type (white bars) and Ste20-related proline–alanine-rich kinase-knock-in (black bars) mice. Blood pressure was assessed using radiotelemetry every day from 1000 to 1100 h, during 17 days (before and after aldosterone infusion). Each bar represents the mean ± standard error of approximately 550 measures of each mouse of each day. *n* = 5 mice for each group. ^∗^*P* less than 0.05 vs. Ste20-related proline–alanine-rich kinase-knock-in.

We also analyzed the effect of aldosterone administration and a high-salt diet on the BP of wild-type and SPAK-KI mice. Figure 3 shows the mean for BP in the basal state and after 14 days of aldosterone administration, similar to the analysis showed in Fig. 1. SBP in wild-type mice increased from 109 ± 7.2 to 126 ± 3.8 mmHg, that is an increment of about 15 mmHg. In contrast, in the SPAK-KI mice, the observed increase was from 105 ± 2.7 to 143 ± 3.4 mmHg, for a difference of about 38 mmHg. Similar difference was observed for the mean BP. Thus, opposite to the observations for AngII, SPAK-KI mice were more sensitive than wild-type mice in response to aldosterone.

**FIGURE 4:**
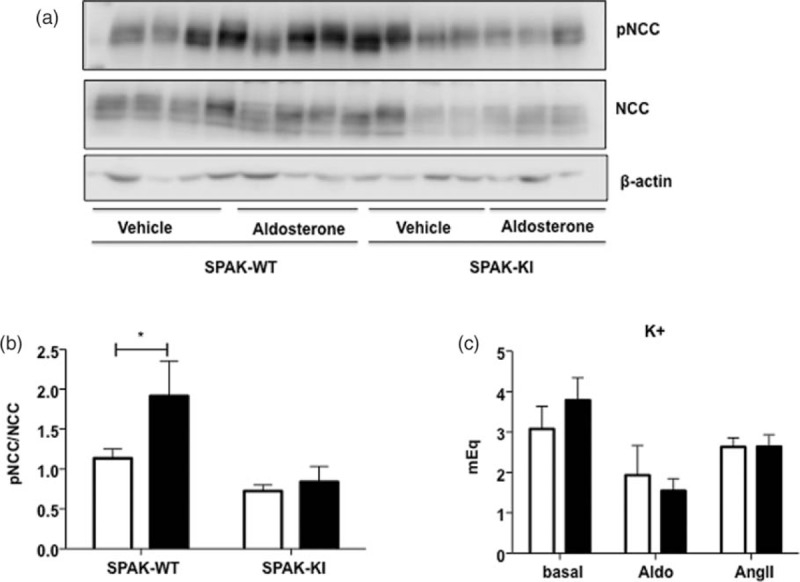
Aldosterone infusion increased the pNa–Cl cotransporter/Na–Cl cotransporter ratio in wild-type mice, but not in Ste20-related proline–alanine-rich kinase-knock-in mice. (a) Representative western blot for total Na–Cl cotransporter, phosphor-Na–Cl cotransporter and β-actin of proteins extracted from the kidney of wild-type or Ste20-related proline–alanine-rich kinase-knock-in mice infused with vehicle or Aldosterone, as stated. (b) Densitometric analysis of two independent western blot for total Na–Cl cotransporter and phosphor-Na–Cl cotransporter, expressed as the pNa–Cl cotransporter/total Na–Cl cotransporter ratio after β-actin normalization. Open bars indicate vehicle infusion, and closed bars indicate aldosterone infusion. (c) Plasma potassium levels after 13-day treatment with aldosterone or angiotensin II in STE20-proline alanine-rich kinase-WT and Ste20-related proline–alanine-rich kinase-knock-in mice. *n* = 5. Open bars show STE20-proline alanine-rich kinase-WT mice and close bars show Ste20-related proline–alanine-rich kinase-knock-in mice. ^∗^*P* less than 0.05 vs. Ste20-related proline–alanine-rich kinase-knock-in basal.

As expected, we observed an increase in the pNCC/total NCC ratio in the wild-type mice (Fig. 4a and b) that was likely due to the hypokalemia induced by aldosterone infusion (Fig. 4c). In contrast, the increased in pNCC did not occur in the SPAK-KI mice treated with aldosterone, despite a similar reduction in plasma potassium, supporting, as has been previously shown, that with no lysine kinase/STE20-proline alanine-rich kinase (WNK/SPAK) pathway is also required to achieve the NCC phosphorylation due to hypokalemia [19,20].

### Na–Cl cotransporter and epithelial sodium channel involvement in the hypertensive effect of aldosterone in Ste20-related proline–alanine-rich kinase-knock-in

**FIGURE 5:**
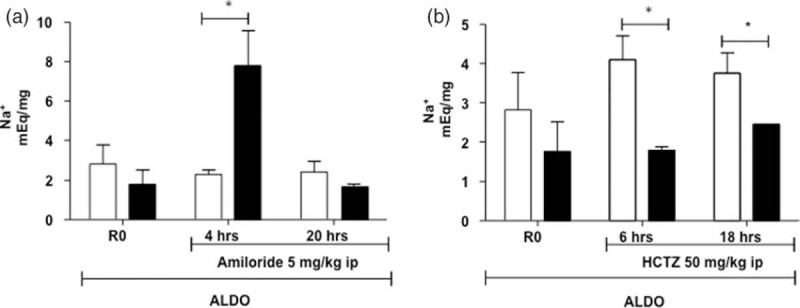
Diuretic challenge with hydrochlorothiazide or amiloride in STE20-proline alanine-rich kinase-WT and Ste20-related proline–alanine-rich kinase-knock-in mice infused with aldosterone and 1% NaCl in the drinking water. Mice were kept in a metabolic cage to determine the basal urinary sodium excretion (adjusted per volume as mEq sodium over mg of creatinine in urine) and the urinary sodium excretion after a single i.p. injection of hydrochlorothiazide (50 mg/kg) or amiloride (5 mg/kg). Bars represent mean ± standard error in wild-type (white) and Ste20-related proline–alanine-rich kinase-knock-in (black), before (R0), 4 and 20 h after amiloride administration, and 6 and 18 h after hydrochlorothiazide administration. *n* = 5. ^∗^*P* less than 0.05 vs. Ste20-related proline–alanine-rich kinase-knock-in at 4, 6 and 18 h.

**FIGURE 6:**
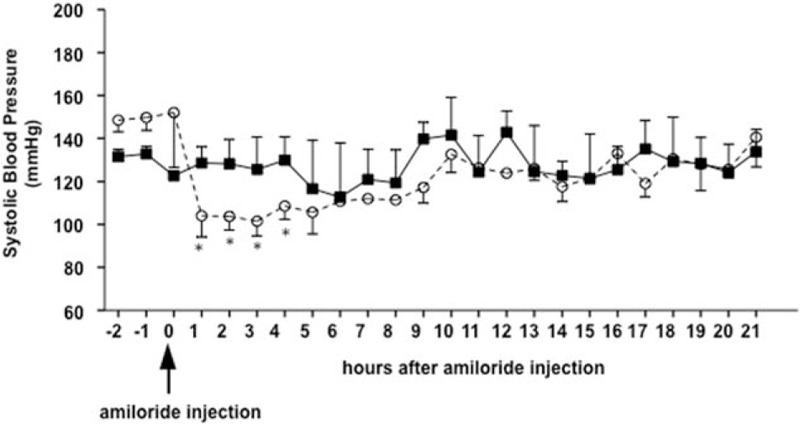
Effect of amiloride injection in blood pressure in aldosterone-infused and 1% Na–Cl Ste20-related proline–alanine-rich kinase-knock-in and STE20-proline alanine-rich kinase-WT mice. Closed squares indicate wild-type mice, and open circles indicate Ste20-related proline–alanine-rich kinase-knock-in mice. By telemetric measured, the response to a single doses of amiloride was tested on 1% Na–Cl-aldosterone-infused Ste20-related proline–alanine-rich kinase-knock-in and STE20-proline alanine-rich kinase. The blood pressure was recorded continuously 2 h before and 12 h after amiloride administration. Each point represents the mean ± standard error of approximately 550 measurements equivalent to 1 h of recording for each mouse. *n* = 5 mice from each group. ^∗^*P* less than 0.05 vs. Ste20-related proline–alanine-rich kinase-knock-in.

To analyze the activated pathway associated with the effect of aldosterone, we used the wild-type and SPAK-KI mice infused with aldosterone and measured the diuretic response to a single injection i.p. of hydrochlorothiazide or amiloride, which are well known blockers of the NCC and the epithelial Na^+^ channel, epithelial sodium channel (ENaC), respectively. As shown in Fig. 5, the natriuretic response to amiloride was significantly higher in the SPAK-KI mice than in the wild-type mice. In contrast, the response to the thiazide diuretic was higher in the wild-type mice than in the SPAK-KI mice. These results suggest that the hypertensive effect of aldosterone in SPAK-KI mice was associated with ENaC activation, rather than NCC activation. We then proceeded to further analyze the role of ENaC in the development of hypertension in the SPAK-KI mice. We tested the effect of amiloride administration in BP after 10 days of aldosterone infusion in both SPAK-KI and wild-type mice. During the first 5 h after i.p. injection of amiloride, a marked reduction in SBP was observed in the SPAK-KI group (140–105 mmHg) but not in the wild-type mice (Fig. 6). This effect was attenuated in the following hours, consistent with the marked decrease in amiloride-induced natriuresis observed after the initial hours of dosage. Nevertheless, a lower SBP was maintained compared with the baseline measurements registered during aldosterone infusion without amiloride. This phenomenon was not observed in the wild-type group. The amiloride injection on SPAK-KI and wild-type mice infused with AngII did not show any effect on BP (Supplementary Fig. 1).

## DISCUSSION

It is known that AngII-induced increase in arterial BP is due to the intrarenal effects of this peptide hormone [2] and several lines of evidence suggest that NCC activation could be responsible, at least partly, for the increased salt reabsorption that precedes the development of hypertension in mice infused with AngII [2]. We have proposed that the AngII effect on the NCC requires the integrity of the WNK-SPAK pathway. By using *Xenopus laevis* oocytes as an expression system, we observed that the activation of NCC by AngII requires the presence of WNK4 [15]. In mpkDCT cells, AngII induced an increase in the phosphorylation of both the SPAK and the NCC [15,21]. Then, we demonstrated that the SPAK phosphorylation induced by a low-salt diet or AngII in wild-type mice was not observed in the WNK4 knockout mice, suggesting that the presence of WNK4 is required for AngII to induce SPAK–NCC phosphorylation [16]. Supporting that AngII effects on DCT require the presence of WNK4, it was observed that AngII, via the PKC pathway, induces KLHL3 phosphorylation in the serine 433 precluding the effect of KLHL3–CUL3 complex on WNK4 and thus, preventing WNK4 ubiquitylation and its destruction [22]. Furthermore, the disruption of the SPAK-NCC cascade via crossing WNK4^D561A/−^ mice (which recapitulate a FHH phenotype) with SPAK^−/−^ mice has been proven to correct the hypertensive and hyperkalemic phenotype associated with NCC hyperactivity [23]. Most recently, we have shown that AngII via PKC phosphorylates WNK4 in key residues increasing the activity of the kinase toward SPAK and NCC [17]. Given these lines of evidence, we used SPAK-KI mice to assess the role of the WNK4–SPAK–NCC pathway in the AngII-induced increase in BP [8].

Our results suggest that, indeed, the AngII-induced hypertension is at least partly dependent on the NCC activation via the SPAK pathway. AngII infusion using a minipump with pressor dose was associated with increased phosphorylation of the NCC in wild-type mice. In contrast, the absence of SPAK activity precluded the phosphorylation of NCC by AngII. Radiotelemetry measurements of BP revealed that AngII-induced hypertension was partially blunted in SPAK-KI mice. The BP still increased, indicating that there are other pathways in the kidney through AngII induces hypertension in addition to the activation of SPAK-NCC. One possibility is that the activation of ENaC plays a role in this response to AngII, because this hormone also increases the activity of this channel [5,24,25]. In addition, AngII also has positive effects on the proximal tubule Na^+^ : H^+^ exchanger, NHE3 [26], as well as in the expression and cleavage of ENaC, both in the cortex and in the medulla which might also be important for salt reabsorption in this setting [27].

Another potential mechanism could implicate blood vessels. It has been suggested that WNK–SPAK pathway is capable to regulate contractibility of the vessels. It is known that NKCC1, that is a target of SPAK, is expressed in vascular smooth muscle cells. Bergaya *et al.*[28], showed that reduction of WNK1 expression in the WNK1^+/−^ prevented phenilephrine-induced vasocontraction of aorta rings and mesentery vessels. However, AngII-induced vasocontraction was similar between control and WNK^+/−^ mice, suggesting a specific alpha-adrenergic activation when the pathway is incomplete [3,28]. Zeniya *et al.*[29] showed that KLHL2, homologue of KLHL3, is present in aorta and vascular smooth muscle cells and AngII diminished its expression and augmented WNK3 expression [29], also suggesting the participation of WNKs in vascular tone. In addition, the SPAK null mice that express a Gitelman-like phenotype (hypotension, hypokalemia and alkalosis) exhibits a decrease in the phosphor-NKCC1 in blood vessels [30] and reduced response to phenylephrine, suggesting that activation of NKCC1 by SPAK may play a role on vasoconstriction. This would explain the differences in BP in the initial days of AngII treatment, during which the effect of AngII on BP might not be entirely attributed to NCC activation. The observation that AngII-induced hypertension is significantly blunted in the absence of SPAK activity supports the proposal that strategies preventing SPAK–NCC interactions could be a new therapeutic avenue for hypertension [31].

In contrast to the observations made with the AngII infusion in the current study, we observed a significant increase in BP in response to the combination of aldosterone and high salt-diet in the SPAK-KI mice. The absence of aldosterone-induced hypertension in the wild-type mice might be explained by the C57BL/6J genetic background of the SPAK-WT and SPAK-KI mice, because C57BL/6J mice are often resistant to aldosterone/salt treatment [32]. The SPAK-KI mice, however, developed a significant increase in BP. After aldosterone infusion, we observed a significantly higher response to amiloride-induced natriuresis accompanied by marked decrease in arterial BP in SPAK-KI mice. In contrast, the response to hydrochlorothiazide was higher in the wild-type mice than in the SPAK-KI mice, consistent with the lower expression of NCC and pNCC in the SPAK-KI mice. In fact, the expected increase of pNCC during aldosterone infusion due to the development of hypokalemia was observed in the wild type, but not in the SPAK-KI mice, indicating that NCC response to changes in serum potassium also requires the integrity of the WNK/SPAK pathway.

Our observations thus suggest that aldosterone-induced hypertension in SPAK-KI mice is mostly associated with the activity of the ENaC in the collecting duct. This is supported by the observations of Rafiqi *et al.*[9], who demonstrated that the expression of all three ENaC subunits was increased in SPAK-KI mice under normal serum aldosterone levels, both in regular and low-salt diet. Of note, due to the decreased expression of NCC and increased expression of ENaC, SPAK-KI mice are thus resistant to AngII-induced hypertension, but particularly sensitive to aldosterone-induced hypertension. As a conclusion, the disruption of the WNK–SPAK pathway attenuates the AH and NCC phosphorylation induced by AngII, while it enhances the aldosterone-induced hypertension mediated by ENaC.

## ACKNOWLEDGEMENTS

We thank the veterinarian Mónica Guevara for her help in the breeding of the SPAK-KI mice colony and Rosalba Perez-Villalba for the help in performing western blots.

The work was supported by grant 091415 from the Wellcome Trust to D.R.A. and G.G. and grant no. 165815 from the Mexican Council of Science and Technology to G.G. D.R.A. is supported by UK Medical Research Council (grant MC_UU_12016/2). L.G.C.-P. was supported by a postdoctoral scholarship from DGAPA-UNAM-Mexico.

The work was partially presented at 2013 Renal Week of the American Society of Nephrology in Atlanta, Georgia, USA and the 2014 Experimental Biology Meeting in San Diego, California, USA.

### Conflicts of interest

There are no conflicts of interest.

## Supplementary Material

Supplemental Digital Content

## Reviewers’ Summary Evaluations

### Reviewer 2

The significance of the present study lies on the differential novel role of WNK-SPAK pathway in hypertension. The disruption of the WNKSPAK pathway attenuates the arterial hypertension and NCC phosphorylation induced by angiotensin II, while it enhances the aldosterone-induced hypertension mediated by ENaC. The weaknesses of the study lie in the lack of exploring deep mechanistic and see whether the reduction in blood pressure is associated with vascular and/or heart structure and function improvement.

### Reviewer 3

The authors investigate whether the WNK/SPAK (with no lysine kinases/Ste20-related proline-alanine-rich kinase) is required for angiotensin II induced arterial hypertension using SPAK knock-in with mutation of threonine 243. This mutation prevents activation of SPAK (SPAK and SPAK-2) by WNKs, decreasing the effect of SPAK on the thiazide-sensitive Na–Cl cotransporter. They provide convincing evidence that angiotensin II dependent hypertension is partially dependent on this pathway whereas aldosterone-induced hypertension depends on epithelial sodium channel activation. The study provides novel mechanistic insights as to how angiotensin II-mediated signaling contributes arterial hypertension but clinical translation may be limited given the availability of thiazides.
