# Supplementary material for: Disruption of the with no lysine kinase–STE20-proline alanine-rich kinase pathway reduces the hypertension induced by angiotensin II
Source: J Hypertens. 2017 Sep 14;36(2):361–7. doi: 10.1097/HJH.0000000000001554 (PMC5757652; doi:10.1097/HJH.0000000000001554)

Supplemental figure 1. The effect in blood pressure of amiloride injection in AngII infused mice. The blood pressure was recorded continuously 2 hours before and 12 hours after amiloride administration. Each point represents the mean ± standard error of approximately 550 measurements equivalent to one hour of recording for each mouse. n=5 mice from each group. p=NS


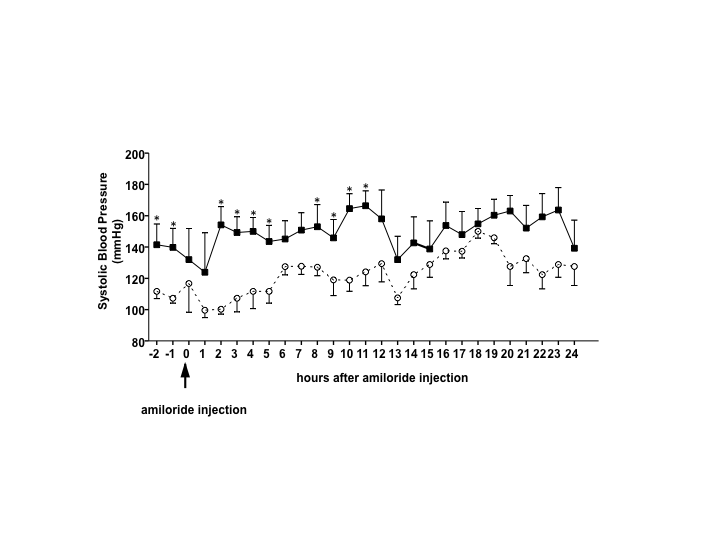

Supplement: Supplemental Digital Content [file jhype-36-361-s001.docx]
